# Comment on “Delivering cellular and gene therapies to patients: solutions for realizing the potential of the next generation of medicine”

**DOI:** 10.1038/s41434-021-00228-y

**Published:** 2021-02-08

**Authors:** Liz Bell

**Affiliations:** 1 EdAd Ltd, Cheltenham, UK; 2 ALP Synergy Ltd, Chepstow, UK

As someone who has worked in the research, education and innovation sector for over 25 years, and particularly on issues connected to the biomedical sciences and bioengineering and their implications for health innovation, the paper “Delivering Cellular and Gene Therapies to Patients: Solutions for realising the potential of the next generation of medicine” [1] offers some profound insights about the future of national healthcare in the 21st century. It looks at the issue of new technologies challenging existing ways of doing things, within delivery and funding frameworks which were designed, and indeed evolved, to deliver healthcare in the 20th century.

The paper draws out the complex issues and challenges posed by new gene and cell based therapeutic approaches in the new era of highly personalised medicine, to the current drug development and treatment infrastructure. It highlights possible solutions that might be explored to make this workable within a predominantly private, insurance based, US healthcare system. However it is not just gene and cell based therapies that are going to challenge this system, 21st century medicine is facing a potential avalanche of new technological approaches from enterprising bioengineers and other biomedical researchers which are set to transform the practice of medicine as we know it.

This leads us to so many issues that need to be addressed by policy makers and other stakeholders. The research underpinning such new developments is high risk and high cost, with uncertain successes and financial returns for the individuals and organisations involved. How can it best be funded and incentivised? New appropriate regulatory frameworks and testing regimes need to be developed to ensure that the products are safe for clinical use, a substantial challenge for policy makers when the technologies are so diverse and their potential applications so personalised. Then as the paper points out, their effective and safe delivery to patients substantially challenges the existing business models of private healthcare funders and providers, including requiring the provision of ongoing training support for physicians and clinicians. The paper gives relevant stakeholders some potential leads that could be followed up to start to adapt business models and practices, but substantial additional research, consultation and deliberation will be needed to make this work.

The elephant in the room is that these technologies may be used only for patients who can afford them, increasing the divide between rich and poor people’s medicine, with the needs of disadvantaged people severely neglected. The characteristics of such new technologies will challenge all current healthcare system models, but particularly complex private ones. Publicly funded and organised systems such as the UK’s NHS, which can work with policy makers, researchers and regulators, centrally purchase medicines, technologies and services, and reorganise hospitals and clinics to provide them to citizens free at the point of delivery, may ultimately make much more effective use of them. The internal economics of such a publicly funded system can more readily adapt, as for example new effective treatments for cancer reduce costs in other parts of the system such as the need for expensive surgery and ongoing drug and other treatments for patients. So, adoption of effective new treatments can ultimately reduce the overall cost to the taxpayer in a publicly funded system, thereby further incentivising their adoption. Good news for the companies and research organisations developing them. Good news for health service providers and patients.

I suspect that adaptation of existing business practices within a predominantly private system will turn out to be an interim measure. The longer term solution will be for government policy makers in all countries, to rethink the overall structure and funding for delivery of effective and equitable 21st century national healthcare systems.
